# Investigate the relationship between cell-phone over-use scale with depression, anxiety and stress among university students

**DOI:** 10.1186/s12888-022-04419-8

**Published:** 2022-12-02

**Authors:** Shima Hashemi, Firoozeh Ghazanfari, Farzad Ebrahimzadeh, Saeed Ghavi, Afsaneh Badrizadeh

**Affiliations:** 1 Student Research Committee, Lorestan University of Medical Sciences, Khorramabad, Iran; 2 Department of Epidemiology, Faculty of Health, Ilam University of Medical Sciences, Ilam, Iran; 3 Department of Psychology, Faculty of Human Sciences, Lorestan University, Khorramabad, Iran; 4 Nutritional Health Research Center, Lorestan University of Medical Sciences, Khorramabad, Iran; 5 Department of Epidemiology and Biostatistics, Faculty of Public Health, Birjand University of Medical Sciences, Birjand, Iran; 6 Social Determinants of Health Research Center, Lorestan University of Medical Sciences, Khorramabad, Iran

**Keywords:** Mental health, Cell phone over use, Stress, Anxiety, Depression, Students

## Abstract

**Background:**

Cell phones have increased dramatically as a new communication technology in the modern world. This study aimed to determine the relationship between cell phone over use scale with depression, anxiety and stress among university students in Khorramabad, Iran.

**Methods:**

In this descriptive-analytical and cross-sectional study, 212 students were randomly selected from the Lorestan University of Medical Sciences by a combination of stratified and clustered random sampling. Data were collected by two standard questionnaires including, Cell-phone Over-use Scale (COS) and Depression, Anxiety and Stress (DASS-21) and were analyzed using SPSS V.22.

**Results:**

Based on the results, 72.2% of the students were exclusively male, which a majority of them were in age of 21–23 years (46.2%), and 92.5% were single. Based on the multiple linear regression and after adjustment for the confounding effect, there was a significant relationship between cell phone over use scale on student’s stress (*t* = 2.614, *P* = 0.010), and student’s anxiety (*t* = 2.209, *P* = 0.028); however there was not a significant relationship between cell phone over use scale on student’s depression (*t* = 1.790, *P* = 0.075).

**Conclusions:**

Harmful use of cell phones can aggravate psychological disorders such as anxiety, stress and depression and by controlling this factor can increase the level of mental health and improve the quality of life in students.

**Trial registration:**

Lorestan University of Medical Sciences. ID: IR.LUMS.REC.1397-1-99-1253.

## Introduction

Cell phone is one of the most important inventions that has changed the world communications. Mobile phones have become as an public industry communication in 1980s [1]. 72% of Americans have a smartphone, and the global average is 43%, however, the harmful use of smartphones can have devastating effects on everyone [2]. The use of mobile phones and other informational and communicational technologies has become as an important part of people’s lifestyle [3, 4]. Although the growth of technology and communications in various fields, has accelerated work and reduced distances and is considered as an opportunity, although it can also be a threat to its users and cause problems on their health [5, 6].

Technology addiction can be defined as a subset of addictive behaviors. Many researchers believe that the use of mobile phones leads to the formation of a type of addiction that is just as destructive as drug addiction, alcohol, overeating, immoral relationships, computer games, the Internet, etcetera [7, 8]. A huge number of communication tools users included young people. Like other types of addiction, communication dependence is associated with symptoms such as anxiety, depression, mood swings, obsessive thoughts, withdrawal, and the breakdown of social relationships. On the other hand, as people’s relationships in the virtual world increase, their relationships in the real world decrease; and in some cases, excessive use of this device causes academic performance reduction, conditioning, and expulsion from university [9].

Due to the increasing use of new communication technologies in the world, there are some concerns about constant exposure to radiofrequency signals from mobile phones and their base stations which could adversely affect human health [10].

On the other side, Covid-19 pandemic has spread rapidly worldwide with an increased use of cell phones for educational purposes. Recent studies showed that the use of smart phones among different age groups has grown significantly in epidemics and has caused problems such as mobile addiction [11, 12]. Different studies showed that there is a significant relationship between the use of phone with anxiety and insomnia [13].

Based on the previous studies, residents living around the antennas showed some symptoms such as headache, anxiety, depression, and fatigue [14–17]. Also, according to researches, excessive use of communication devices such as mobile phones, especially among young people, is associated with psychological problems, including depressive symptoms [18]. In addition, students with depressive moods, use cell phones and other communicational tools by the means of spending time [19].

In Thomee’s study, the average excessive use of cell phone among adolescents was associated with their poor sleep patterns and depressive symptoms [20]. The results of Azoki’s study showed that young people who use text messaging in an extreme and addictive manner, will experience high levels of impulsivity, loneliness and social anxiety as well [21].

Also, reduction in the size of social relationships, social isolation and increasing the feeling of loneliness in people who use cell phones frequently, will be one of the most likely consequences of excessive use of mobile phones [22]. People who are addicted to mobile phones feel depressed, confused and isolated without this tool [23]. According to researches, anxiety is also associated with cell phone addiction. The study of Hawi et al. showed that anxiety was positively associated with cell phone addiction [24]. In another study by Sapacz et al. was found that using cell phone was associated with social anxiety [25].

Nowadays, there is a significant growth in the use of mobile phones, while there is not enough consideration to the psychological and social effects of long-term use of this communication’ tool. Youth mental health is one of the most important topics in the psychology and sociology issues. Most students have different uses of mobile phones and one of the problems that employments should focus on, is poor academic performance or poor academic achievement. Despite the increase in number of cell phone users, the mobile phone has not been the subject of serious and scientific research yet. So this study was aimed to investigate the relationship between harmful cell phone usage with depression, anxiety and stress among university students.

## Methods

This descriptive-analytical cross-sectional study was conducted among Lorestan University of Medical Sciences students in 2019. Students studying in the faculties of Lorestan University of Medical Sciences were included after their conscious satisfaction about the study’s content. This study was approved by Lorestan University of medical Sciences. Written informed consent and verbal agreement was taken from all participants. The confidentiality principle was maintained so that there was no need to mention the names of the individuals in the questionnaires, and it was assured that the information was just provided to the researcher and used in the study. All methods were carried out in accordance with relevant guidelines and regulations or declaration of Helsinki.

Sampling was done only in different faculties of Lorestan University of Medical Sciences located in Khorramabad city. Sampling method was a combination of stratified and clustered random sampling; in this way, each faculty was considered as a stratum and within each faculty stratum there was a sub- stratum (field of study, gender, and degree). There were also sub-stratums within each clusters (entries of different years).

Finally, a field of study from each faculty was selected and one to two entries from each specific field were selected randomly (for example, one entry from lower grade students, e.g. semesters one, two, three, and four, and the next entry from undergraduate students, e.g. semesters fifth, sixth, seventh and eighth) and then, proportional to size systematic random sampling was deployed in each sub-stratum. Totally 212 students were collected as the sample size based on the formula below (considering 1.5 of design effect):

$${\displaystyle \begin{array}{l}n=\frac{{\left({z}_{1-\alpha /2}+{z}_{1-\beta}\right)}^2\left[{p}_1\left(1-{p}_1\right)+{p}_2\left(1-{p}_2\right)\right]}{{\left({p}_1-{p}_2\right)}^2}\simeq 141\\ {} final\kern0.17em sample\kern0.17em size=1.5\times n\simeq 212\end{array}}$$

Student’ demographic information tool and two standard questionnaires were used in this study.

1. The demographic information questions were including: Gender, age, marital status, faculty, major, GPA, educational level, students’ residence, parents’ residence, father’s educational level, mother’s educational level, household size, and monthly household income.
2. Depression Anxiety and Stress Scale-21 (DASS-21): DASS-21 [26] with 21 items and three subscales (7 items for each subscale) was used. This instrument measures the prevalence of depression, anxiety, and stress signs and symptoms during the past weeks. The items of the scale are rated on a four-point Likert scale, with the score for each item ranging from 0 (“does not apply to me at all”) to 3 (“applies to me most of the time”). The subscale scores were calculated by summing the scores of the individual items, and the maximum sum for each subscale is 21; higher scores represent higher psychological distress. The normal score for severity of each subgroup is (0–9) for depression, (0–7) for anxiety, and (0–14) for stress and very sever scores vary to (> 28) for depression, (> 20) for anxiety, and (> 33) for stress. The original study of [26] reported the high reliability of DASS-21, Cronbach’s Alpha coefficients for depression, anxiety, and stress were reported as 0.91, 0.84, and 0.90, respectively. Henry and Crawford [27] found this scale to have good concurrent and discriminant validity as well as excellent internal consistency (α = .93). In this study, the reliability of dimensions of depression, anxiety, and stress was 0.83, 0.79, and 0.77.
3. Cell-phone Over-use Scale (COS): The harmful factors caused by cell phone use were asked. This questionnaire was developed by Gennaro et al. (2007), and the reliability of this scale was determined by Cronbach’s alpha internal consistency method on Spanish female and male students were reported 0.85. This scale has 21 items. The scale is graded based on a six-point Likert (never (0), almost never (1), sometimes (2), often (3), almost always (4), and always (5). Subjects with scores above 75 were rated as excessive users and less than 25 were assigned to be sparingly users [28, 29]. In this study, the validity was good and the reliability was 0.88.

Data were analyzed via SPSS software version 22 at significance level of 0.05. For descriptive purposes, frequency distribution table and some statistical measures such as, mean and standard deviation and for normality checking, one-sample Kolmogorov-Smirnov test was deployed. One-way ANOVA test and independent t-test were used for assessment the relationship between demographic variables with cell phone over use, stress, anxiety and depression scores, on the other hand. Furthermore, Pearson’s correlation coefficient test was deployed in order to evaluating the linear relationship between cell phone over use, stress, anxiety and depression scores. In multivariable modeling, three separate multiple linear regression models were used. In all three models, independent variable was cell phone over use score and also, some demographic variables were considered as confounders. Those demographic variables, which had *P*-values less than 0.250, both with independent variable (cell phone over use score) and dependent variable (any three dimensions of mental health) were considered as, potentially confounding variable and their effect on dependent variable were adjusted.

## Results

According to the results, 72.2% of students who participated in this study were exclusively male, which a majority of them were in age of 21–23 years (46.2%), whom 92.5% were single. Based on their parental educational level, 36.3% of their fathers and 20.3% of their mothers had academic education. About 65.1% of the students lived in dormitories. In terms of resistance, 92.5% were living in urban and 7.5% in rural area. Additionally 72.6% were studying in associated degree and Bachelor of Science while the rest 27.4% were studying in Master of Science and PhD. degree. All other socio-demographic characteristics of the participants are provided in (Table 1).

**Table 1:** Frequently distribution of student’s demographic variables

Variables	Categories	Number (Percent)
Gender	Female	153 (72.2)
	Male	59 (27.8)
Age (years)	18–20	87 (41.0)
	21–23	98 (46.2)
	≥ 24	27 (12.7)
Marital Status	Single	196 (92.5)
	Married	16 (7.5)
Faculty	Medicine	50 (23.6)
	Dentistry	13 (6.1)
	Health & Nutrition	55 (25.9)
	Paramedical sciences	64 (30.2)
	Nursing & Midwifery	30 (14.2)
Major	Operating room technology	14 (6.6)
	Anesthesia	15 (7.1)
	Health information technology	14 (6.6)
	Medical emergencies	9 (4.2)
	Radiology technology	13 (6.1)
	Nutritional sciences	14 (6.6)
	Public Health	12 (5.7)
	Occupational health engineering	15 (7.1)
	Environmental health engineering	13 (6.1)
	Dentistry	12 (5.7)
	Medicine	35 (16.5)
	Laboratory sciences	16 (7.5)
	Nursing	16 (7.5)
	Midwifery	14 (6.6)
GPA	< 15	34 (16.0)
	15–16.99	109 (51.4)
	≥17	69 (32.5)
Educational level	Associated Degree & BSc	154 (72.6)
	MSc & PhD	58 (27.4)
Students’ residence	Dormitories	138 (65.1)
	Non- dormitories	74 (34.9)
Parents’ residence	Urban	196 (92.5)
	Rural	16 (7.5)
Father’s educational level	Illiterate to high school	76 (35.8)
	Diploma	59 (27.8)
	Academic Education	77 (36.3)
Mother’s educational level	Illiterate to high school	89 (42)
	Diploma	80 (37.7)
	Academic Education	43 (20.3)
Household Size	< 4	20 (9.4)
	4–5	105 (49.5)
	≥6	87 (41)
Monthly household income	< 200 $	41 (19.3)
	200–350 $	67 (31.6)
	≥350 $	104 (49.1)

Table 2, shows the absolute and relative frequency of cell phone over use variables, stress, anxiety and depression among university students.

**Table 2:** Absolute and relative frequency of cell phone over use variables, stress, anxiety and depression among university students

Variable	Category	Number (Percent)
**Cell phone over use**	Mild	20 (9.4)
	Moderate	184 (86.8)
	Severe	8 (3.8)
**Stress**	Mild	204 (96.2)
	Moderate	8 (3.8)
**Anxiety**	Mild	162 (76.4)
	Moderate	27 (12.7)
	Severe	23 (10.8)
**Depression**	Mild	184 (86.8)
	Moderate	23 (10.8)
	Severe	5 (2.4)

The results of one-way ANOVA showed that there was a statistically significant meaning between faculty (*p* = 0.004), major (*p* = 0.050), and GPA (*p* = 0.038) in terms of cell-phone over-use. Although, there was no statistically significant meaning between gender, age category, marital status, educational level, father’s educational level, mother’s educational level, monthly household income, student’s residence, parent’s residence, and household Size (*p* > 0.05).

It is also showed that there was just a statistically significant meaning between faculty (*p* = 0.040), in terms of the average score of stress. Although, there was no statistically significant meaning between GPA, gender, age category, marital status, educational level, father’s educational level, mother’s educational level, monthly household income, student’s residence, parent’s residence, and household Size (*p* > 0.05).

There was a statistically significant meaning between faculty (*p* = 0.004), and GPA (*p* = 0.015) in terms of the average score of anxiety. Although, there was no statistically significant meaning between gender, age category, marital status, educational level, father’s educational level, mother’s educational level, monthly household income, student’s residence, parent’s residence, and household Size (*p* > 0.05).

The results also showed that there was just a statistically significant meaning between faculty (*p* = 0.006) in terms of the average score of depression. Although, there was no statistically significant meaning between GPA, gender, age category, marital status, educational level, father’s educational level, mother’s educational level, monthly household income, student’s residence, parent’s residence, and household Size (*p* > 0.05) (Table 3).

**Table 3:** Comparison between Cell phone over use score, Stress, Anxiety, and Depression with student’s demographic variables

Demographic variable	Category	Cell phone over use score		Stress score		Anxiety score		Depression score	
		mean ± s.d.	*P*-value	mean ± s.d.	*P*-value	mean ± s.d.	*P*-value	mean ± s.d.	*P*-value
Gender	Male	49.8 ± 17.7	0.070	6.5 ± 3.6	0.835	5.5 ± 3.5	0.137	5.2 ± 4.6	0.150
	Female	45.3 ± 15.6		6.6 ± 3.8		4.8 ± 3.2		4.4 ± 3.4	
Age (years)	18–20	46.9 ± 15.8	0.974	6.6 ± 3.6	0.743	5.2 ± 3.5	0.426	4.4 ± 3.8	0.793
	21–23	46.4 ± 16.2		6.7 ± 3.8		4.6 ± 3.2		4.8 ± 3.9	
	≥24	46.3 ± 18.8		6.1 ± 4.0		5.3 ± 3.0		4.4 ± 3.4	
Marital Status	Single	46.6 ± 16.1	0.933	6.6 ± 3.8	0.300	5.0 ± 3.3	0.852	4.7 ± 3.8	0.548
	Married	46.2 ± 18.3		5.6 ± 2.7		4.8 ± 3.6		4.1 ± 3.4	
Faculty	Medicine	48.8 ± 15.1	0.004	7.9 ± 3.6	0.040	6.3 ± 3.3	0.004	6.2 ± 4.3	0.006
	Dentistry	50.7 ± 17.0		5.5 ± 3.1		3.1 ± 2.7		3.3 ± 3.5	
	Health & Nutrition	40.8 ± 15.6		5.8 ± 3.8		4.4 ± 2.8		3.8 ± 3.2	
	Paramedical sciences	45.3 ± 14.6		6.5 ± 4.0		5.0 ± 3.7		4.8 ± 4.0	
	Nursing & Midwifery	54.0 ± 18.9		6.2 ± 3.1		4.2 ± 3.3		3.7 ± 2.9	
GPA	< 15	49.6 ± 15.0	0.038	7.4 ± 3.5	0.178	6.4 ± 3.9	0.015	5.7 ± 4.7	0.172
	15–16.99	48.1 ± 17.2		6.1 ± 3.8		4.8 ± 3.3		4.4 ± 3.5	
	≥17	42.5 ± 14.6		6.8 ± 3.8		4.4 ± 3.0		4.4 ± 3.7	
Educational level	Associated Degree & BSc	46.3 ± 16.6	0.754	6.5 ± 3.8	0.610	4.8 ± 3.3	0.176	4.4 ± 3.6	0.304
	MSc & PhD	47.1 ± 15.5		6.8 ± 3.5		5.5 ± 3.3		5.1 ± 4.4	
Monthly household income	< 200 $	48.5 ± 16.1	0.683	6.7 ± 3.9	0.926	4.9 ± 3.5	0.806	5.3 ± 4.4	0.114
	200–350 $	46.0 ± 16.4		6.4 ± 3.3		5.2 ± 3.1		5.0 ± 3.9	
	≥350 $	46.1 ± 16.3		6.6 ± 3.9		4.9 ± 3.4		4.1 ± 3.4	
Father’s educational level	Illiterate to high school	44.7 ± 15.5	0.188	6.2 ± 3.7	0.088	4.9 ± 3.1	0.088	4.6 ± 3.6	0.685
	Diploma	49.8 ± 15.3		7.5 ± 3.7		5.7 ± 3.3		5.0 ± 3.7	
	Academic Education	45.9 ± 17.5		6.2 ± 3.7		4.5 ± 3.5		4.4 ± 4.1	
Mother’s educational level	Illiterate to high school	45.9 ± 16.1	0.813	6.4 ± 3.6	0.707	4.9 ± 3.1	0.870	4.8 ± 3.6	0.882
	Diploma	46.6 ± 15.0		6.8 ± 3.9		5.1 ± 3.5		4.5 ± 3.9	
	Academic Education	47.8 ± 19.0		6.4 ± 3.8		4.8 ± 3.5		4.6 ± 4.1	
Students’ residence	Dormitories	45.3 ± 15.6	0.129	6.4 ± 3.8	0.553	5.0 ± 3.3	0.659	4.7 ± 3.8	0.512
	Non- dormitories	48.9 ± 17.3		6.8 ± 3.7		4.8 ± 3.3		4.4 ± 3.9	
Household Size	< 4	42.0 ± 14.7	0.396	6.8 ± 3.3	0.892	5.2 ± 3.1	0.852	4.7 ± 3.5	0.990
	4–5	47.4 ± 17.1		6.6 ± 3.8		5.0 ± 3.5		4.6 ± 4.1	
	≥6	46.5 ± 15.5		6.4 ± 3.9		4.8 ± 3.1		4.6 ± 3.5	
Parents’ residence	Urban	46.9 ± 16.2	0.314	6.7 ± 3.8	0.052	5.0 ± 3.4	0.295	4.8 ± 3.8	0.057
	Rural	42.6 ± 16.7		4.8 ± 2.8		4.1 ± 2.5		2.9 ± 2.8	

The results of the Pearson correlation coefficient analyses between psychological variables including cell phone over use, stress, anxiety and depression are shown in (Table 4). A strong correlation was found between Cell phone over use score and Stress score (*r* = 0.212, *p* = 0.002), whereas the correlation between cell phone over use score and anxiety score was (*r* = 0.193, *p* = 0.005), and the correlation between cell phone over use score and depression score was (*r* = 0.153, *p* = 0.026).

**Table 4:** Pearson correlation between study variables

Variables	Correlation coefficient	***P***-value
**Cell phone over use score**		
**Stress score**	0.212	0.002
**Anxiety score**	0.193	0.005
**Depression score**	0.153	0.026
**Stress score**		
**Anxiety score**	0.643	< 0.001
**Depression score**	0.608	< 0.001
**Depression score**		
**Anxiety score**	0.650	< 0.001

Based on the multiple linear regression and after adjustment for the confounding effect, there was a significant relationship between cell phone over use scale on student’s stress (t = 2.614, *P* = 0.010); so that by increasing in each unit of cell phone over use score, the student’s stress score increases about 0.040 units. In other words, by increasing in every 25 units of cell phone over use score, the student’s stress score increases about 1 unit.

Also after adjustment for the confounding effect, there was a significant relationship between cell phone over use scale on student’s anxiety (t = 2.209, *P* = 0.028); so that by increasing in each unit of cell phone over use score, the student’s anxiety score increases about 0.031 units. In other words, by increasing in every 33 units of cell phone over use score, the student’s stress score increases about 1 unit.

Finally after adjustment for the confounding effect, there was not a significant relationship between cell phone over use scale on student’s depression (t = 1.790, *P* = 0.075); so that by increasing in each unit of cell phone over use score, the student’s anxiety score increases about 0.028 units. In other words, by increasing in every 36 units of cell phone over use score, the student’s stress score increases about 1 unit (Table 5).

**Table 5:** Adjusted effect of Stress, Anxiety, and Depression on Cell phone over use, by using three different multiple linear regression

Dependent variable	Model coefficients							Model fit	
	Unstandardized		Standardized	95.0% Confidence Interval for B		t	***P***-value	R^**2**^	***P***-value
	B	Std. Error	Beta	Lower Bound	Upper Bound				
Stress score	0.040	0.015	0.175	0.010	0.070	2.614	0.010^*^	0.119	< 0.001
Anxiety score	0.031	0.014	0.150	0.003	0.058	2.209	0.028^**^	0.129	0.003
Depression score	0.028	0.016	0.119	−0.003	0.059	1.790	0.075^***^	0.130	< 0.001

^*^Adjusted for father’s educational level, parents’ residence, GPA and faculty

^**^Adjusted for gender, father’s educational level, educational level, GPA and faculty

^***^Adjusted for gender, monthly income, parents’ residence, GPA and faculty

## Discussion

The improper use of cell phone has increased dramatically nowadays. Because electromagnetic waves are used to transmit data via cell phones, some concerns have been raised because wave’s negative effects also affect human public health. The effects of cell phone over use on anxiety, depression and stress are some of these concerns that can be assessed by laboratory methods, epidemiological studies and standard questionnaires [30].

The results of this study showed that there was no relationship between cell phone over use with stress, anxiety, depression, gender, age, marital status, educational level, father and mother educational level, students’ residence, parent’s residence, household size, however there was a significant relationship between faculty with cell phone over use, stress, anxiety, and depression (*p* < 0.05). Based on this study, there was a significant relationship between GPA with cell phone over use and anxiety (*p* < 0.05); however there was no significant relationship between GPA, stress and depression.

One of the identified variables associated with cell phone over use is depression. Depression is a psychological disorder that causes marked changes in mood, outlook, perfectionism, thinking ability, activity, and bodily processes such as sleep, energy, and appetite [31]. Depression is a disorder that is caused by a variety of factors including biological, social, psychological, behavioral and environmental pressures and stresses. Cell phone addiction is one of the factors that has caused and exacerbated various mental disorders such as anxiety and depression recently [32]. Many previous studies showed that there is a positive relationship between depression and cell phone over use among students. In other words, it can be said that as depression increases, their cell phone overuse increases as well, which is consistent with previous studies [18, 33]. In this study there was a significant positive correlation between depression and stress (*r* = 0.608), and depression and anxiety (*r* = 0.650).

In short, students experience more negative emotions such as depression, anxiety, and stress because they are in a sensitive developmental period. Therefore, to reduce negative emotions and psychological experiences, they use fun activities such as cell phones to reduce these stresses temporarily and divert their focus from their perceived stress. Reducing negative emotions following cell phone use leads to intensify spending more time with their cell phone in order to receive more reinforcements and reduce negative emotions, and this behavior gradually becomes as an addictive behavior [34].

The study of the Tas [35] showed that depression and anxiety have significantly predicted cell phone addiction, which was consistent with the results of Kil et al. [36], and Volungis et al. [37], who observed positive and significant correlation between cell phone addiction and anxiety, depression and stress. Zanjani et al. [38], indicated that the more depression among students, the higher rate of cell phone addiction.

Based on the present study, there was a significant positive correlation between anxiety and cell phone over use (*r* = 0.193), and anxiety and stress (*r* = 0.643). Anxiety has a strong correlation with the cell phone over use, which is one of the disorders that people usually suffer from [2]. In addition, the results of this study showed that there was a significant relationship between cell phone over use and anxiety and psychological problems such as depressive symptoms, which is consistent with other studies [30, 39].

Hawi’s study also found that cell phone over use through anxiety is indirectly related to family relationships and there is also a positive correlation between cell phone over use and anxiety [24]. Samaha et al. [40] stated that the higher the smartphone addiction, the higher the level of anxiety, and the higher the level of anxiety, the more increases cell phone over use, which leads to a vicious cycle. In other words, a stressor that raises the level of anxiety may also increase the harmful use of cell phones. In explaining the relationship between excessive cell phone usage and the level of anxiety and depression among students, researchers have pointed to the increased stress caused by cell phone waves and the effect of these waves on brain activity. Excessive employment in cell phone calls causes a lot of stress, worries and anxieties among young people. These disorders are such that a kind of mental and neurological disease is defined based on it and the sufferer feels his cell phone ring several times during the day [41, 42]. Physical reactions such as headache or the sensation of heat caused by prolonged use of a cell phone can also be stressful and lead to concerns about the potential dangers of using electromagnetic devices [20].

Based on different studies, the use of mobile devices has increased during Covid-19 pandemic [43]. The heavy usage of this technology may have a negative impact on students’ physical and mental health and also it may cause some other problems such as depression and anxiety [44].

One of the limitations of the present study was that it was performed only among the students of one university and also does not include engineering, basic sciences and humanities students and should take more care in generalizing the findings to other individuals and students. It would be interesting to provide more comprehensive studies to other provinces especially with more completed statistical communities in future. The results of this study on anxiety, stress and depression were based on participants’ self-expression and there was no clinical evidence in this regard. On the other hand, the extent of cell phone over use did not have an objective measurement tool and was evaluated on the basis of self-declaration. Another limitation of this study was the use of correlation scheme to show the relationship between variables. Therefore, in order to clarify the relationship between the variables, longitudinal studies in this field are needed.

## Conclusions

The main purpose of this study was to find out the relation between Cell-phone Over-use Scale and various components like stress, anxiety and depression. There was a significant positive correlation between Cell-phone addiction and stress, anxiety and depression. So, smartphone addiction should be prevented in young adults to save them from getting behavioral problems such as stress, anxiety and depression. Future studies can be done using larger sample size and on the different age group of people.

## Data Availability

All data generated or analyzed during this study are included in this published article.
